# The Week's Work

**Published:** 1910-10-08

**Authors:** 


### THE WEEK'S WORK.

### THE KING EDWARD MEMORIAL PROPOSALS.

The Lord Mayor's Committee of the Mansion House Fund for providing a memorial in London to King Edward VII. has printed and circulated the suggestions which it has received as to the form the memorial should take. From that list we give all those likely to interest our readers. It will be seen what a large proportion those relating to hospital endowment, research, and care for the sick bear to the whole category. A special ward in each hospital to be called King Edward VII. Ward is the one, perhaps, with the greatest chance of adoption. Others are a Sanatorium for Consumptives; a Free Home for Incurables of the Lower Class; the erection of "Houses of Recovery" for Hospital Patients, and the establishment of an Institution for Cancer Research, though in view of the large sums recently given to research of this type this suggestion presents obvious difficulties. Then there are proposals for Homes for Soldiers Invalided from Consumption; for an Imperial Institute of Hygiene and Tropical Medicine; for an Annuity Fund for Disabled Nurses; for increased support to the League of Mercy, together with a scheme for Extirpating Tuberculosis in Man and Animals; a Fund or Hospital for Soldiers' Widows; the Nationalisation or "Councilisation" of all Medical, Surgical, and Dental Practice; a Home for Incurables on the non-voting system; a Great Free Hospital for Cancer Patients; and finally an appeal for the support and re-naming of King's College Hospital. Most of these suggestions propose to identify the memorial rather too exclusively with specialised interests to make them escape the objection of not being national enough in scope. Still it is noteworthy that one person out of every twelve who has offered suggestions for King Edward VII.'s Memorial has proposed to identify the late King's name still further with some branch or other of medical or hospital work—a striking tribute to the force of King Edward's example. Of other suggestions of general interest we may add that the most ambitious is Lord Eversley's proposal for a monumental chapel associated with Westminster Abbey on a scale like the famous Henry VII. Chapel, some beautiful designs for which were made by Mr. Pearson, R.A., a few years ago. The most popular, perhaps, is the proposal to refront Buckingham Palace.

### THE GLASGOW CONFERENCE.

The first annual conference of the British Hospitals' Association, held at Glasgow last week, may be regarded as a signal success upon which the officers of the Association, and everyone responsible for the work of the meeting, are to be heartily congratulated. The quality of the papers read was excellent, and the discussions that followed their reading were in all cases instructive and elucidative of many interesting aspects of the problems under review. On another page will be found an account of the essential features of the work done, and in next week's issue and in succeeding numbers, we hope to publish the various papers read at the Congress *in extenso*. Each paper will be amplified by a report of the discussion that followed it, and in this way we hope to give our readers, who have not had the opportunity of attending the meetings, a full and correct summary of the transactions. The Association owes a debt of gratitude to the officials who have worked so energetically to make the first conference a success, to the Corporation of Glasgow and many private friends for their generous hospitality extended to the organisation during the sittings of the conference, and last, but by no means least, to the members who contributed papers and took part in the discussions.

### THE MEDICAL EXHIBITION.

The annual exhibition, organised by the *British and Colonial Druggist*, which was opened on Monday last in the Royal Horticultural Hall, Vincent Square, Westminster, is a yearly event which should interest the institutional worker no less than the practitioner. It is true that the exhibition appeals more prominently to the latter, and the display of new drugs and new appliances is apt to strike the hospital secretary with amazement and trepidation when he considers his already increasing drug bill. But there are many points of real interest, from an institutional aspect, to be seen at the exhibition, not the least interesting being the ward furniture shown by several firms, the hydro-therapeutic apparatus, and some of the foodstuffs in concentrated form. We understand that the exhibition is only open to qualified medical men, and that the public is rigidly excluded. In a professional museum, such as this is, that is an admirable proviso, but we have no doubt that an exception will be made on behalf of such institutional workers as are *bona-fide* members of a hospital staff and who desire to inspect the collection. We would suggest to the organisers that next year's exhibition might be made more comprehensive still by including in it more of those subjects that appeal to the hospital man, and that special invitation cards might be issued to hospital officials. For the exhibition is a valuable one, and the most complete of its kind in the country.

### HOSTELS IN THE COUNTRY.

Of late years there has arisen a class of institution intermediate between a convalescent station and an ordinary boarding-house. This is the country hostel, the primary object of which is to afford a temporary asylum for those who, although not invalids in the ordinary sense of the term, are in need of a period of rest and repose amid hygienic and invigorating surroundings. Such institutions are practically extensions of the city hostel, though they serve a much wider population and have a much larger range of usefulness. In the majority of cases they are voluntary charitable institutions, receiving inmates from specified districts, although there is no general rule that limits the attendance at a particular hostel to boarders from one district. The charges made for a week's board and lodging at such institutions is usually a little in excess of what is charged at a convalescent home, but they are still very low: so low, in fact, that unless these institutions have a very large patronage, they are not self-supporting. In some cases the hostel is open all the year round: in others it is open merely during the summer months, it having been found that there is hardly any demand for it in winter.

### THE GREEN LADY HOSTEL.

We have recently had the opportunity of inspecting one of these institutions, the Green Lady Hostel at Littlehampton, and we would specially call attention to the letter from the Honorary Treasurer of that institution which appears in this issue of The Hospital. The Green Lady is an excellent little home, situate in a rural and bracing environment, and thoroughly to be recommended in all respects. The building is practically new, and although plain and unpretentious is well suited for its purpose, and has so far answered admirably. This hostel is one of the "summer class," that is to say, it closes in winter time owing to the fact that it draws its patrons largely from the London factories where the recognised holiday time is in the spring. The advantages of such a "holiday" home are incalculable. To the institutional worker it should appeal if only for the fact that if we had more such homes the out-patient population—and the in-patient for that matter—would be largely diminished. At present, hospital officials find it sometimes rather difficult to advise an out-patient who is anæmic, out of condition, but not sufficiently ill to warrant a definite diagnosis of a lesion, which is a justifiable excuse for claiming the privileges of treatment in the hospital convalescent home. Such a patient cannot afford to go to an ordinary rest home; a country or seaside boarding-house is even more out of the question, and, unfortunately, we have not yet an organisation that does for adults what the Invalid Aid Association does so excellently for children—boarding out. In such a contingency it is as well to bear in mind that these hostels exist, and that they cater very well for this class of patient. Like all institutions that depend for their support on public generosity, they are in want of funds. In the case of the Littlehampton Hostel, for which Miss Montague pleads, those who are desirous of helping it may be fully assured that their donations will serve a useful purpose, and will be the means of assisting this institution to carry on what is after all highly necessary, and, in some respects, pioneering work.

### THIS WEEK'S DRUG MARKET.

Business has been very fair during the week, and most of the alterations in price are in an upward direction. The price of cocaine has again been advanced owing to the scarcity of the crude product. As was expected, the syndicate controlling the output of santonin has added 1s. 6d. per lb. to the value of the article. Terpin hydrate is dearer. Cream of tartar has again advanced in price, and a good steady business continues to be done. Both Russian and Spanish ergot are dearer. Digitalis leaves are scarce, and prices are advancing. The long-expected advance in the price of potassium bromide and other bromides has at last been announced; the advance is at the rate of about 2d. per lb., but makers are only selling for prompt delivery, and a further advance is probable. Menthol is again dearer, and still higher prices are expected. Japanese peppermint oil is also advancing. Opium is unchanged in value, and no further alterations have been made in the price of morphine and codeine. Sugar of milk is dearer. A further advance in the price of glycerine is expected to take place at any moment. As was anticipated, the price of quicksilver has been reduced by 5s. per bottle, but no reduction has been announced in the price of mercurial salts. The price of saccharine has been reduced. Rhubarb is selling at what may be considered low prices.

